# E3 ubiquitin ligases: styles, structures and functions

**DOI:** 10.1186/s43556-021-00043-2

**Published:** 2021-07-30

**Authors:** Quan Yang, Jinyao Zhao, Dan Chen, Yang Wang

**Affiliations:** 1 Second Affiliated Hospital, Institute of Cancer Stem Cell, Dalian Medical University, Dalian, 116044 China; 2 Department of Pathology, First Affiliated Hospital, Dalian Medical University, Dalian, 116044 China

**Keywords:** E3 ligases, Ubiquitination, 26S proteasome degradation, Cancer progression, Therapeutics, PROTACs

## Abstract

E3 ubiquitin ligases are a large family of enzymes that join in a three-enzyme ubiquitination cascade together with ubiquitin activating enzyme E1 and ubiquitin conjugating enzyme E2. E3 ubiquitin ligases play an essential role in catalyzing the ubiquitination process and transferring ubiquitin protein to attach the lysine site of targeted substrates. Importantly, ubiquitination modification is involved in almost all life activities of eukaryotes. Thus, E3 ligases might be involved in regulating various biological processes and cellular responses to stress signal associated with cancer development. Thanks to their multi-functions, E3 ligases can be a promising target of cancer therapy. A deeper understanding of the regulatory mechanisms of E3 ligases in tumorigenesis will help to find new prognostic markers and accelerate the growth of anticancer therapeutic approaches. In general, we mainly introduce the classifications of E3 ligases and their important roles in cancer progression and therapeutic functions.

## Introduction

Almost all proteins in cells and some of the extracellular proteins are constantly updated through degradation and replacement with newly synthesized proteins. The degradation of proteins is mainly through two major pathways: autophagy and ubiquitin proteasome system (UPS), both of which are essential for maintaining cellular homeostasis[1]. Autophagy is a crucial adaptive mechanism to deal with different cellular stresses via degrading excessive or abnormal proteins in cells mediated by lysosomes [2]. The UPS is a cascade reaction and an important way for short-lived, misfolded, and damaged proteins degradation [3]. As reported, the UPS can regulate degradation of over 80% proteins in cells and its dysregulation has been revealed in most hallmarks of cancer [4]. Above all, E3 ligases are the important part of the UPS and can provide regulatory specificity [5]. E3 ubiquitin ligases regulate the last step of the enzymatic cascade, which also consists of ubiquitin activating enzymes (E1s) and ubiquitin conjugating enzymes (E2s). E3 ligases can selectively attach ubiquitin to lysine, serine, threonine or cysteine residues to the specific substrates [6]. The process of attaching ubiquitin and ubiquitin-like proteins to cellular proteins is called ubiquitylation, which plays a vital role during posttranslational modification (PTM) [7]. As reported, the ubiquitin-proteasome degradation pathway is one of the most important mechanisms for controlling the levels of protein expression. Furthermore, ubiquitylation process also has profound effects on the cellular localization, interactions or stability of proteins [8, 9]. In this review, we will firstly introduce the various types of E3 ligases family. Then, we will explain the biological functions and molecular mechanisms of E3 ligases in cancer development. Finally, we summarize the novel therapeutic role of E3 ligases in cancer treatment.

## The types and cascade process of ubiquitination

**Fig. 1:**
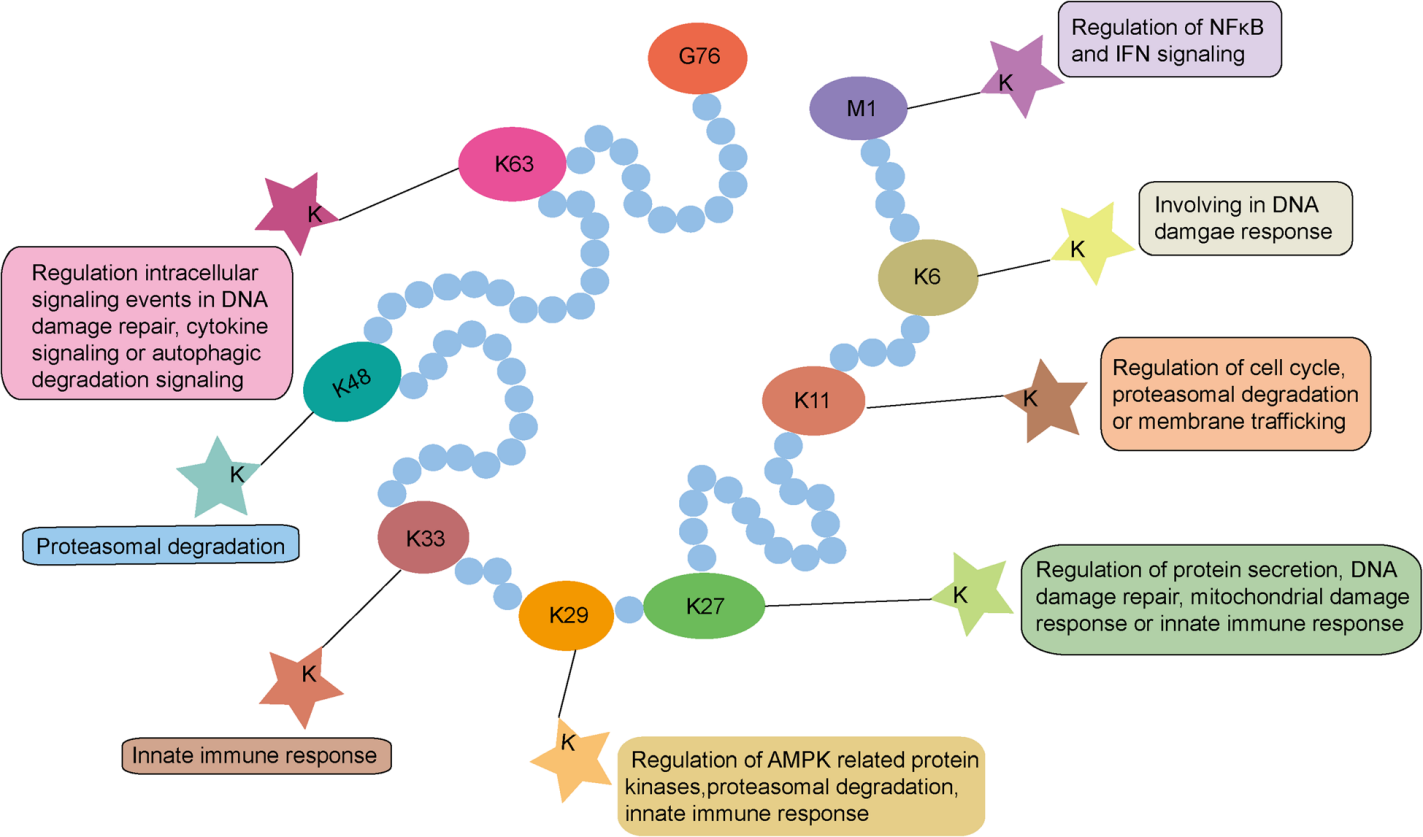
Different ubiquitin linkage types and their functions. Ubiquitin (Ub) a small protein consists of 76 amino acids highly conserved among all eukaryotes. Ubiquitin is characterized by its 7 lysine sites (K6, K11, K27, K29, K33, K48, K63) and N-terminal methionine1 (Met1) site, which are also functional sites. Due to these specific linkage sites, ubiquitination can be classified into different styles and then perform distinct biological functions. And ubiquitin attaches to target substrate through identifying its lysine (K) site

Ubiquitin (Ub) consists of 76 amino acids that is highly conserved among all eukaryotes [10]. The essential features of ubiquitin protein are the seven key lysine sites (K6, K11, K27, K29, K33, K48, and K63) and its N-terminus. As reported, no matter in the intracellular environment or the extracellular reaction system, every lysine site of ubiquitin and the N-terminal methionine 1 (Met1) site can respectively form different ubiquitin linkage types [11]. In general, there are eight main kinds of ubiquitin linkage types and each of them performs distinct physiological functions [12]. During these linkage types, K48 and K63 linked ubiquitination are two of the most well studied. K48-linkages are the most abundant ubiquitination chains in biological processes. The main function of K48-linkages is to target substrates for 26S proteasome-mediated degradation [13]. K63-linkages are mainly involved in intracellular signaling events in DNA damage repair, cytokine signaling or autophagic degradation signaling [1, 14, 15]. Besides, the functions of other linkage types have not been studied in much detail till now so that they are referred as ‘atypical’ ubiquitin linkages. K6-linkages have been reported to participate in DNA damage response as well [16]. K11-linkages are implicated to regulate cell cycle, proteasomal degradation or membrane trafficking. K11-linkages can even influence innate immune response by targeting innate immune factors for degradation [17–19]. K27-linkages are associated with protein secretion, DNA damage repair and mitochondrial damage response involved by the E3 ligase Parkin [20, 21]. K27-linkages are also important activators of the innate immune response. For example, the E3 ligase RNF185 targets cGAS and AMFR targets STING for K27-linked ubiquitination, both leading to proinflammatory and antiviral response [22, 23]. K29-linkages have been reported to play a role in proteasomal degradation, innate immune response and regulation of AMPK related protein kinases [24–26]. K33-linkages are related with the regulation of innate immune response through affecting cGAS-STING- and RLR-induced type I IFN signaling and intracellular trafficking [27, 28]. Met1-linkages also called linear ubiquitin chains have been revealed to be catalyzed by linear ubiquitin chain assembly complex (LUBAC), which consists of two RBR type E3 ligases, HOIL-1 and HOIP [29]. Met1-linkages are capable of promoting the activation of NFκB signaling by targeting NEMO a member of IKK complex that phosphorylates NFκB inhibitor α (IkBα). Meanwhile, Met1-linkage can likewise inhibit type I IFN signaling through mediating NEMO and TRAF3 interaction and then disrupting MAVS-TRAF3 complex (Fig.1) [30, 31]. Furthermore, according to the way ubiquitin acts on its substrates, ubiquitination modifications can be classified into three styles: mono-ubiquitination, multi-ubiquitination, and polyubiquitination. Mono-ubiquitination is defined as the target substrate labeled with a single ubiquitin. And mono-ubiquitination regulates the function of substrates via a nonproteolytic mechanism [32]. Multi-ubiquitination is defined as many different lysine residues of the target substrate are labeled with a single ubiquitin at the same time. However, polyubiquitination is defined as a single lysine residue of the target protein labeled with multiple ubiquitin molecules [33].

**Fig. 2:**
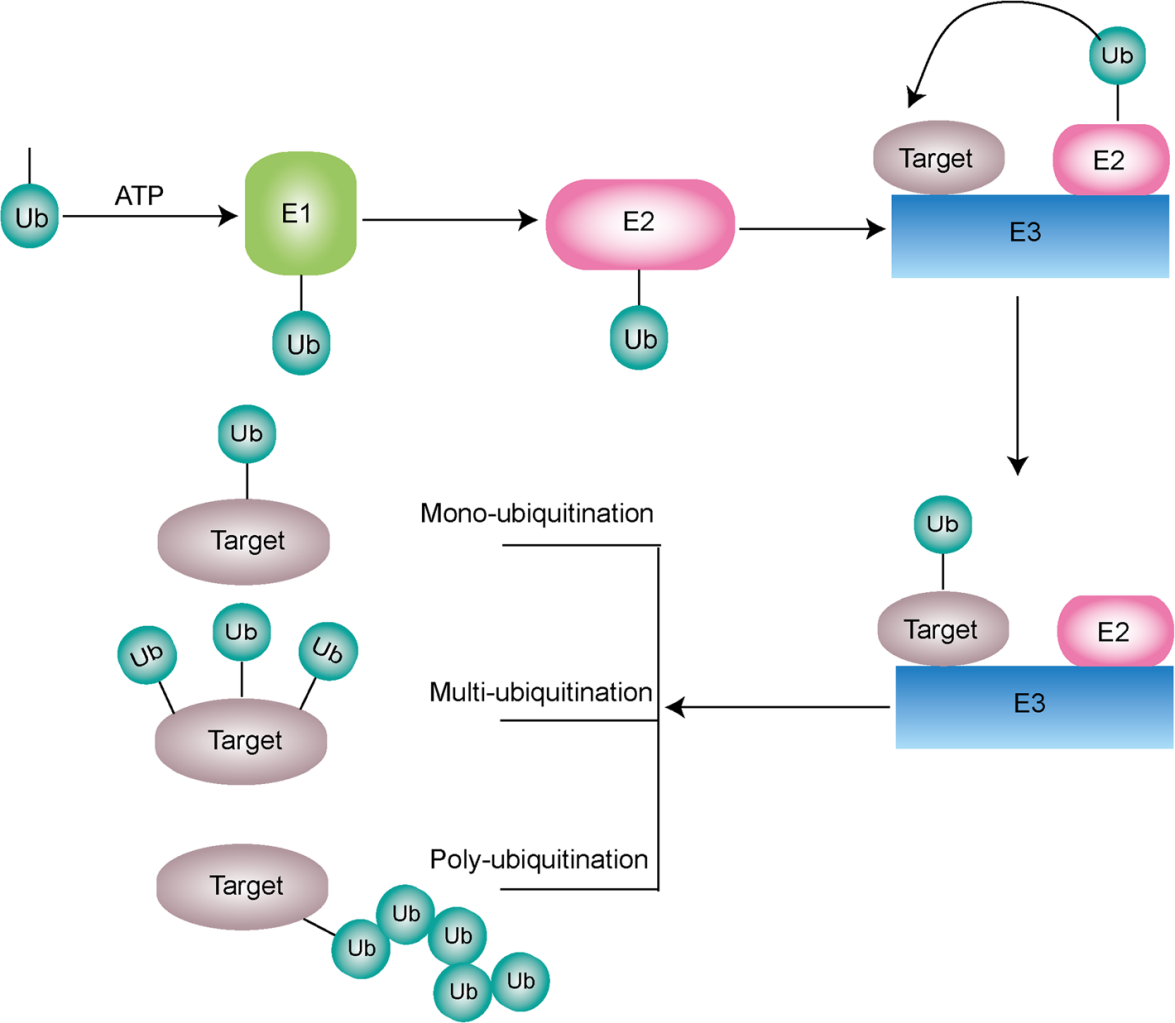
Overview of the cascade process of ubiquitination. Ubiquitination is an important cascade process of posttranslational modification catalyzed by three key enzymes. Firstly, E1 catalyzes the activation of Ub through a thioester bond in an ATP dependent mechanism. Then, activated Ub is transferred to the active-site cysteine residue of an E2. The last step is mediated by an E3 ligase that recognizes the E2 complex and facilitates the transfer of Ub from E2 to the target substrate. Due to the different binding style of Ub on the target substrate, types of ubiquitination modification are divided into three: mono-ubiquitination, multi-ubiquitination and poly-ubiquitination

Ubiquitination is defined as a series of enzymatic cascades consisting of three crucial enzymes, including E1s, E2s, and E3 ubiquitin ligases. Firstly, E1 enzyme catalyzes the formation of a thioester bond between its own active cysteine site and the C terminus of ubiquitin in an ATP-dependent manner. Subsequently, the activated ubiquitin is joined to the active cysteine site of E2 via a thioester bond. Finally, E3 ligase recruits the E2 loaded with the activated ubiquitin. The E3 ubiquitin ligase interacts with both the target substrate and E2 ubiquitin ligase and then catalyzes the transfer of ubiquitin from the E2 to the target substrate directly or indirectly. The ubiquitin is bond to a lysine site on the target substrate by an isopeptide bond (Fig.2) [34, 35].

## The classification and features of E3 ligases

As described previously, not only the increasing amount but also the function of E3 ligases have been proved to play a key role in cancer progression. As E3 ligases can directly bind to substrates and determine the specificity of ubiquitin system, there would be a large number of E3 ligases but only a few E1 and E2 ligases in distinct organisms [36, 37]. According to the difference of structure and function, E3 ligases can be approximately divided into four types: HECT type, U-box type, RING-finger type, RBR type. Interestingly, different types of E3 ligases have low sequence homology and large differences in composition [36].

### HECT E3 ligases

**Fig. 3:**
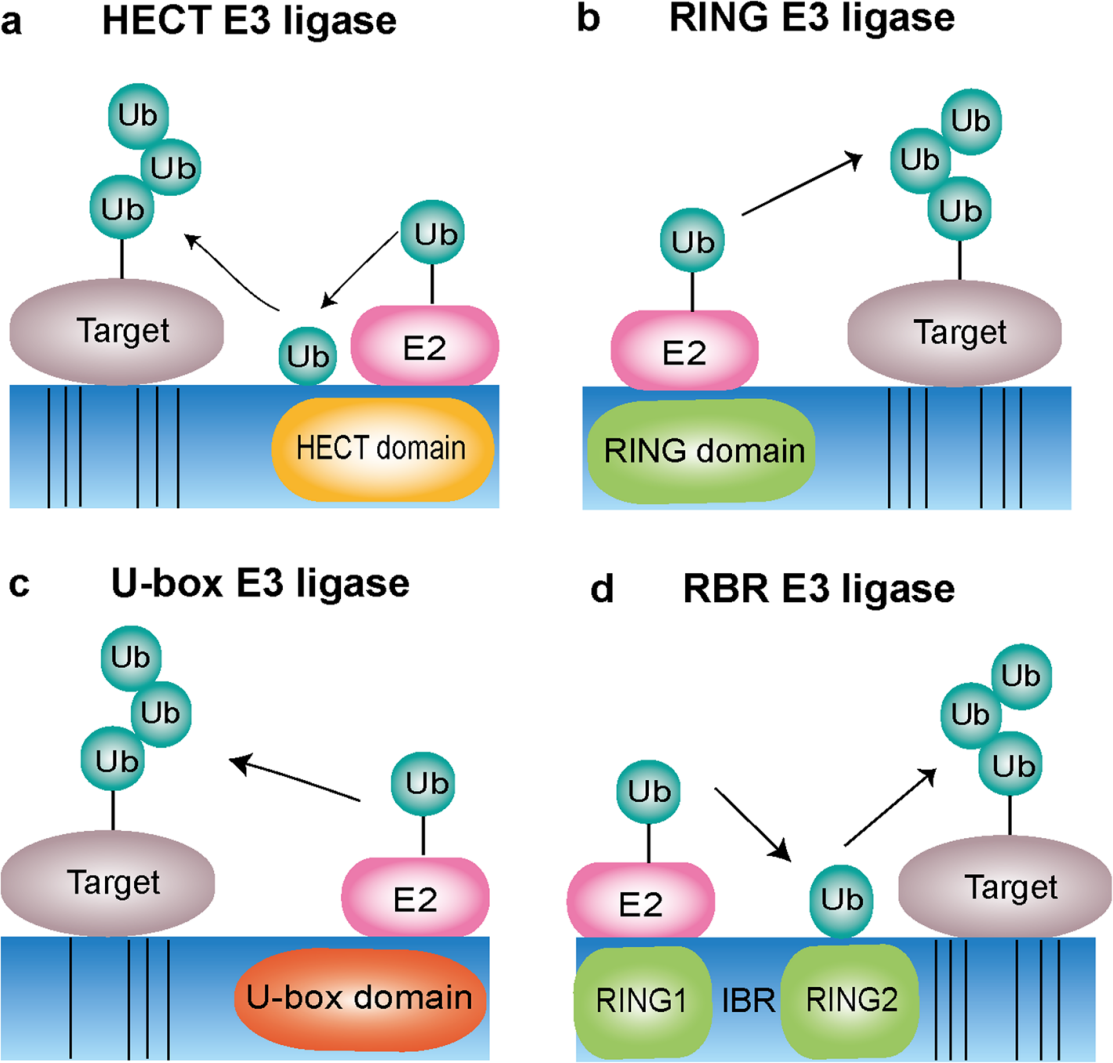
Types of ubiquitination ligases. **a** The HECT type E3 ligases contain the conserved C-terminal HECT domain and the N-terminal consists with different domains depending the specific subtype. HECT type E3 ligases involved ubiquitination process including a two-step reaction: ubiquitin is first carried by E2 ligase binding to the HECT domain and then transferred to a catalytic cysteine on the E3 ligase, the second step is the transfer of ubiquitin from the E3 ligase to the substrate. **b** The RING type E3 ligases are characterized by the presence of a zinc-binding domain called RING at the N-terminal. RING E3s mediate a direct transfer of ubiquitin from E2 ligase to the substrate. **c** The U-box type E3 ligases contain U-box domain at the C-terminal which is responsible for binding the ubiquitin-charged E2 ligase and stimulating ubiquitin transfer. **d** The RBR type E3 ligases consist of two predicted RING domains (RING1 and RING2) separated by IBR domain . RBR E3 ligases catalyzed ubiquitination process involves a two-step reaction where ubiquitin is first transferred to a catalytic RING2 domain on the E3 and then to the substrate

HECT (homologous to the E6AP carboxyl terminus) E3 ligases family is one of the largest and earliest studied E3 ligases [38]. HECT ligases contain a common homologous to E6-associated protein C-terminus (HECT) domain, where the activated E2 ligase can transfer Ub to the active cysteine site before binding to the target substrate. The N-terminal domains are the positions where target substates bind (Fig.3a) [39]. Due to the difference of N-terminal domain, HECT E3 ligases can be classified into three groups: the Nedd4 family (9 members), the HERC family (6 members) and another HECTs (13 members) [36]. In addition to the common HECT C-terminal domain, the Nedd4 subfamily is specialized by the presence of WW and C2 domain that is also well studied. The N-terminal C2 domain can bind a Ca^2+^ and phospholipid, which is not only necessary for targeting proteins to phospholipid membranes, but also can help target substrate proteins for ubiquitination [40–42]. The HERC subfamily is characterized by containing one or more RCC-like domains (RLD) [43]. According to the number of RLDs, HERC subfamily can be further classified into two large and four small HERCs. RLDs have two major functions that can regulate the small GTPase Ran as a guanine nucleotide exchange factor (GEF) and interact with chromatin through histones H2A and H2B [44, 45]. Additionally, there are still many other HECT ligases including E6AP and HUWEI. E6AP plays the founding member and contains a zinc-binding fold named the AZUL (amino-terminal Zn-finger of Ube3a ligase) domain. However, HUWE1 contains a WWE domain and a ubiquitin-associated (UBA) domain, which affects various aspects of cancer development [46, 47].

### RING-finger E3 ligases

RING (really interesting new gene) E3 ligases are the major type of E3 ligases and characterized by their RING domain [6, 48]. There are more than 600 different RING type ligases expressed in human cells [49]. During ubiquitination process, the RING domain of RING E3 ligases binds the E2 conjugation enzyme. Contrary to HECT E3 ligases, the Ub is transferred from the E2 to the substrate directly, bypassing an E3-Ub intermediate (Fig.3b) [50, 51]. RING E3 ligases are divided into two big families: monomeric RING finger and multi-subunit E3 ligases. Monomeric RING E3 ligases not only have the domain for substrate binding and ubiquitination, but also have the function of autoubiquitination, such as COP1, Mdm2, and TRAF6 [52]. Multi-subunit E3 ligases, such as the cullin-RING ligases (CRLs) are a highly diverse class of ubiquitin ligases characterized by several common features. The cullin scaffold includes the N terminus RING-box protein, an adaptor protein, and the C terminus substrate receptor. Another crucial multi-subunit E3 ligases APC/C is assembled of 19 subunits including a RING subunit (Apc11) and a cullin-like subunit (Apc2) [53, 54]. SCF E3 ligases are the largest E3 ligases complex, including Skp1, Cullin1 and F-box proteins [55]. These proteins connect with each other and perform distinct functions. F-box is crucial for the recognition of the substrates. Skp1 is responsible for binding the catalytic core of the SCF complex to the F-box motif. Meanwhile, Cullin1 is necessary for adjusting the connection with other SCF complex components [56, 57]. RING E3s can be also regulated by different modifications, including autoubiquitination, neddylation, phosphorylation, and interaction with small molecules [58].

### U-box E3 ligases

U-box E3 ubiquitin ligases are a relatively small family, which is necessary for controlling the quality of post-translational protein in eukaryotic cells [59]. The C-terminus of U-box E3 ligases contains a conserved U-box domain of about 70 amino acid residues from yeast to humans. The three-dimensional structure of U-box is similar to the RING finger domain that is necessary for the enzymatic activity [60]. The process of U-box E3 ligases catalyzed ubiquitination is defined as that ubiquitin-binding enzyme E2 interacts with U-box ligase through the U-box domain. Subsequently the Ub is directly transferred from E2 to identify the lysine site of substrate (Fig.3c) [61].

### RBR E3 ligases

The newly discovered RING-IBR-RING (RBR) E3 ligases are proved as a unique family of RING-HECT hybrid E3 ligases, which are not the same as RING and HECT types. The RBR E3 ligases are specialized by a conserved catalytic region, including a RING1, a central in-between-RINGs (IBR) and a RING2 domain [62]. RING1 can recruit the E2 loaded with ubiquitin, and RING2 domain contains a catalytic cysteine. The IBR domain can adopt the same fold as the RING2 domain, when lacking the catalytic cysteine residue. Additionally, different RBR E3 ligases also contain specific domains to distinguish from each other. RBR E3 ligases can be involved in intermolecular interactions to keep the proteins in an autoinhibited state. Such state is regulated by different kinds of mechanisms, such as phosphorylation or protein-protein interactions [63]. In analogy with HECT E3 ligases, RBR E3 ligases, such as human homolog of Ariadne (HHARI) and Parkin for example, perform its function through two-step reactions, and the Ub is firstly transferred to a catalytic cysteine site on RING2 and then to the substrates [64, 65]. Although they are generally similar to HECT E3 ligases, RBR ligases tend to ubiquitinate substrates through linear ubiquitin chain, which is a distinct mechanism [58]. Thus, the linkage specificity of RBR E3 ligases suggests a distinct and more striking mechanism (Fig.3d) [66]. The linear ubiquitin chain assembly complex (LUBAC) is a multi-subunit E3 ligases complex consisting of HOIP, HOIL-1L, Parkin and SHARPIN. LUBAC can specifically assemble Met1-linked (also known as linear) Ub chains to modulate NF-κB signaling [67–70].

## The multi-functions of E3 ligases in regulating cancer progression

**Fig. 4:**
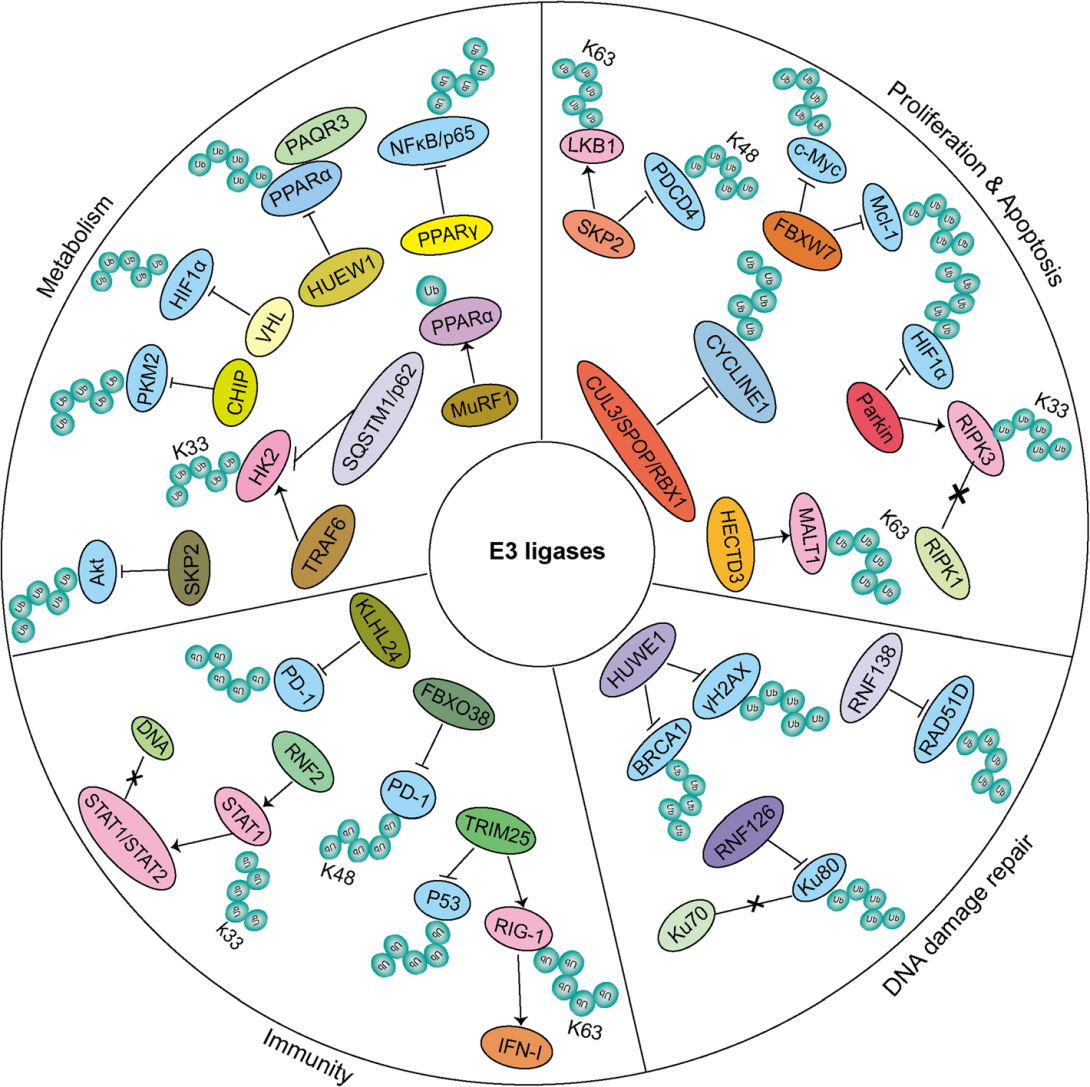
Multi-functions of E3 ligases. Based on the different E3 ligases and their specific substrates, E3 ligases can involve in many different cellular progression such as proliferation, apoptosis, DNA damage repair, immunity and metabolism. We have shown some E3 ligases mentioned in this review and how they function in regulating cancer cell progression. For example, the E3 ligases FBXW7, HECTD3, CUL3/SPOP/RBX1, Parkin, SKP2 are involved in regulating proliferation or apoptosis through targeting proliferation-associated proteins for ubiquitination. E3 ligases HUWE1, RNF126, RNF138 are shown to regulate cancer cell DNA damage repair by targeting specific substrates for ubiquitination. E3 ligases FBXO38, KLHL22, TRIM25, RNF2 regulate cancer cell immune response through promoting ubiquitination of specific substrates. And E3 ligases PPARγ, SKP2, CHIP, VHL, TRAF6, HUEW1,MuRF1 are capable of mediating cancer cell metabolism by targeting related substrates for ubiquitination. Note:we use different background colors to distinguish each E3 ligases. Blue background represents the genes they target for degradation and pink background represnets genes they target for stabilization or others but not degradation. We use × to show the disruption of protein-protein interaction

Ubiquitination modulates cellular functions through maintaining the homeostasis and coping with various stress stimulation. Thus, the dysregulation of ubiquitination process can result in multiple human diseases, especially cancer [71]. E3 ligases are critical for modulating cellular homeostasis owning to their efficient regulation and substrate specificity during the cascade of ubiquitination. Evidently, E3 ligases are significantly involved in cancer progression, such as proliferation, invasion, apoptosis, DNA damage and repair, metabolism, immunity and many other aspects (Fig.4) [33].

### Regulation of cancer cell proliferation, invasion and apoptosis

HECTD3 (Homologous to the E6-associated protein carboxyl terminus domain containing 3) is one of HECT E3 family that contains an N-terminal DOC domain and a C-terminal HECT domain [72]. Many evidences have proved that HECTD3 is a pro-survival protein in several types of cancer. It has been discovered that the overexpression of HECTD3 mainly regulates the K63 but not K48 polyubiquitination, thereby promoting the stabilization of MALT1. The stabilized MALT1 activates CARMA3–Bcl10–MALT1 pathway in angiotensin II receptor-positive breast cancer, leading to the promotion of cancer cell proliferation and invasion [73, 74]. NEDD4 is also a canonical C2-WW-HECT subgroup E3 ligase that has been proved to positively modulate proliferation of cancer cells. The tumor suppressor PTEN is a relevant substrate of NEDD4, whose ubiquitination brings about the degradation or translocation shuttling and erlotinib resistance, eventually contributing to non-small-cell lung cancer progression [75, 76]. SCF E3 ligases are one of the most typical example of multi-subunits-RING type E3 ligases. SCF complex is mainly comprised of RBX1, SKP1, CUL1 and F-box protein family, whose dysregulation is highly related with cancer progression [77]. RBX1 contains the canonical RING finger domain. RBX1 is overexpressed in multiple cancers, thus to modulate the proliferation of gastric cancer cells [78]. Besides, RBX1 is more likely to form a complex with other E3 ubiquitin ligases to show its function. In addition to the SCF complex, RBX1 is also a member of the critical subunits of CUL3/SPOP/RBX1 complex. Importantly, CUL3/SPOP/RBX1 complex is proved to suppress prostate cancer progression by targeting CYCLIN E1 for polyubiquitination degradation [79]. CUL1 is not only the first and most widely studied member of cullin family, but also a necessary scaffolding component of SCF complex to form a catalytic core complex [77]. CUL1 is reported to promote breast cancer cell migration and invasion through inducing relative cytokine gene expression, such as CXCL8 and IL11 [80]. The F-box proteins are specialized by an amino-terminal 40-residue F-box motif, which are able to stimulate the specific ubiquitination of various substrates [81]. FBXW7 is a tumor suppressor extensively studied in different kinds of human cancers, that is deleted or mutated in various cancers [82]. FBXW7 is capable of interacting with Mcl-1, the pro-survival Bcl-2 family member, to facilitate the degradation via ubiquitination in a GSK3 phosphorylation-dependent manner, leading to cancer cell apoptosis [83]. FBXW7 is also the E3 ligase of an important oncogene c-Myc. FBXW7 accelerates the degradation of c-Myc and inhibits the tumor cell proliferation in Adult T-cell Leukemia [82, 84]. Another important F-box protein SKP2 significantly involves in the regulation of cell cycle and proliferation. SKP2 stabilizes LKB1 via K63-linked ubiquitination that is required for the growth of cancer cell. Meanwhile, SKP2 and LKB1 are both overexpressed in late-stage hepatocellular carcinoma [85]. In addition, SKP2 stimulates breast cancer tumorigenesis through K48-linked ubiquitination of the tumor suppressor PDCD4 [86]. As an important multi-subunit RING type E3 ligase, APC/C promotes the transition from metaphase to anaphase during mitosis, which implicates its pivotal role in controlling cellular division and tumorigenesis [87]. Cdc20 and Cdh1 are two essential activators of APC/C that determine the specificity of APC/C to substrates during cell cycle. Cdc20 is highly associated with various cancers. Overexpression of Cdc20 can promote the activation of prostate cancer progression related WNT/ β-catenin pathway by regulating β-catenin [88]. APC/C^Cdc20^ can also regulate apoptosis by targeting Bim, a pro-apoptotic protein, and Mcl-1, a pro-survival protein, for degradation [87]. Furthermore, Cdc20 is able to target the tumor suppressor SMAR1 for polyubiquitination degradation in kinds of cancers, such as breast cancer, cervical and colon cancer [89]. Another APC/C activator Cdh1 characterized as tumor suppressor is highly associated with cancer progression. The highly important function of the E3 ligase APC/C^Cdh1^ is to regulate cell cycle, promoting the transition into G1 through targeting mitotic proteins for degradation [87]. APC/C^Cdh1^ can suppress MEK/ERK oncogenic pathway by targeting BRAF oncogenic kinase for degradation [90]. Importantly, APC/C^Cdh1^ tends to promote cancer cells to adapt to immune response by destabilizing SPOP, as cullin 3-SPOP is the direct E3 ligase to target PD-L1 for degradation. Thus, APC/C^Cdh1^ is able to regulate the expression of PD-L1 indirectly [87, 91]. The RBR type E3 ligase Parkin is highly related with Parkinson’s disease and important in controlling mitochondrial homeostasis and ROS [92]. In addition, Parkin can also function as a tumor suppressor and inactivated in various human cancers. Parkin is reported to ubiquitinate HIF1α for degradation through its lysine 477, eventually inhibiting breast cancer cell migration and invasion [93]. Furthermore, Parkin is capable of blocking the RIPK1−RIPK3 interaction, which is important in modulating necroptosis and AMPK activation by targeting RIPK3 for K33-linked polyubiquitination. Therefore, Parkin tends to prevent inflammation-induced cancer by inhibiting necroptosis and many other promising mechanisms (Table 1) [95, 96].

**Table 1:** Keys E3 ligases involve in the regulation of cancer progression

E3 ligase	Target	Cancer/Cell type	Function	Refs
HECTD3	MALT1	breast cancer	proliferation and invasion	[73, 74]
NEDD4	PTEN	lung cancer	proliferation	[75, 76]
RBX1	CYCLIN E1	prostate and gastric cancer	proliferation	[78, 79]
CUL1	CXCL8, IL8	breast cancer	migration and invasion	[80]
FBXW7	Mcl-1, c-Myc	leukemia	apoptosis, proliferation	[82–84]
SKP2	LKB1, PCDC4, Akt	liver and breast cancer	proliferation, metabolism	[85, 86, 94]
APC/C	Bim, Mcl1, SMAR1, BRAF, SPOP	prostate, breast, cervical and colon cancer	apoptosis, proliferation, cell division	[87–91]
Parkin	HIF1α, RIPK3	breast cancer	migration, invasion, necroptosis	[92, 93, 95, 96]
HUWE1	BRCA1, Mcl1, P53	breast cancer	DNA damage repair	[97–100]
WWP1	P53	breast and prostate cancer	DNA damage repair	[100]
RNF138	RAD51D	breast and ovarian cancer	DNA damage repair	[101, 102]
RNF126	Ku80, PDK1	293T, U2OS cells	DNA damage repair, glycolysis	[103, 104]
BCA2	γH2AX, Rad51	breast cancer	DNA damage repair	[105]
TRIM25	RIG-1, P53	prostate cancer	tumor immunity	[106–110]
FBXO38	PD-1	293T, Jurkat cells	tumor immunity	[111, 112]
C-Cbl	PD-1	colorectal cancer	tumor immunity	[113]
CHIP	PKM2	ovarian cancer	glycolysis	[114]
VHL	HIF1α	renal cancer	glycolysis	[115–117]
TRAF6	HK2	liver cancer	glycolysis	[118, 119]
UFM1	PDK1	gastric cancer	glycolysis	[120]

### Regulation of DNA damage repair

DNA damage and repair systems are extremely important in regulating human biology and disease, especially cancer. DNA damage may directly lead to cell death or gene mutation and even malignant transformation of cells [121]. DNA double-strand breaks (DSBs) can recruit DDR proteins around the break sites and assemble into a highly ordered, dynamic complex for repair. Two major DNA repair pathways are used in eukaryotic cells, including non-homologous end joining (NHEJ) and homologous recombination, as well as branches of these pathways to repair DSBs [122]. It has been reported that the ubiquitination of chromatin around the DSB site also participates in the repair process of DDR, suggesting the possible role of E3 ligases [123]. The HECT type E3 ligase HUWE1 is highly associated with DNA repair through its ubiquitination functions in cancer development [33]. BRCA1 shows great importance in regulating DNA damage repair through homologous recombination (HR), leading to genomic instability in breast and ovarian cancers [97]. HUWE1 mediates DNA repair by promoting H2AX and BRCA1 ubiquitination and degradation, resulting in the suppression of HR-dependent DSB repair that is critical for breast cancer progression [98]. HUWE1 also affects DNA damage responses by regulating the stability of polymerases Pol β and Pol λ during base excision repair. Pol β can be mono-ubiquitinated at Lys-41, 61, and 81 by HUWE1 and subsequently degraded through the E3 ligase CHIP catalyzed poly-ubiquitination [99]. Interestingly, HUWE1 can target both the anti-apoptotic protein Mcl1 and the tumor suppressor p53 for ubiquitination degradation in different conditions. HUWE1 has been implicated to target Mcl1 for ubiquitination in response to DNA damage [100]. Conversely, HUWE1 is incapable of regulating the expression of p53 in response to DNA damage [124]. The E3 ligase WW domain-containing ubiquitin E3 ligase 1 (WWP1) may modulate the development of breast and prostate cancer by affecting the activity of p53 in response to DNA damage [100]. The RING type E3 ligase RNF138 is composed of an N-terminal RING finger domain and a putative C-terminal ubiquitin interaction motif [101]. RNF138 plays vital roles in DNA damage repair by binding to DNA damage sites with the zinc finger domain, thereby ubiquitinating key repair factors. Such ubiquitination could accelerate DNA end resection and promote ATR-dependent signaling and DSB repair by HR pathway [125]. Hence, RNF138 may contribute to cancer cell survival in response to DSB-induced agents [126]. RNF138 can ubiquitinate the RAD51D protein for degradation, affecting the homologous recombination (HR)-mediated DNA repair [101]. And the inactivation mutation of RAD51D contributes to breast and ovarian cancer development [102]. The expression of RNF138 can regulate cellular response to different DNA-damage agents, especially the recruitment of RPA, CtIP, Exo1 and Blm to DNA damage sites, thereby controlling HR repair [126, 127]. The RING type E3 ligases RNF126 and breast cancer associated gene 2 (BCA2) both play important roles in DNA damage repair and cancer development. RNF126 targets Ku80 for polyubiquitination and dissociates Ku70/Ku80 from DNA, accelerating NHEJ-mediated DNA repair in 293T and U2OS cells [103]. BCA2 regulates DDR through interacting with many DDR related proteins such as γH2AX and Rad51 [105]. Since both of RNF126 and BCA2 could regulate DNA damage repair and cancer development, they may be the promising targets for cancer therapy (Table 1) [105].

### Regulation of immunity

Mammalian immune system functions as an essential defense to monitor homeostasis, to resist the invasion and infection of pathogens, and even to eliminate abnormal cells. Therefore, immune system plays vital roles in response to tumorigenesis [128]. Recently, tumor immunology has been a research hotspot and therapeutic target, which is defined as body's immune response to tumor and the mechanism of tumor cell escape immune effect [129]. Accumulating evidence suggests that E3 ligases can regulate innate and adaptive immunity through ubiquitination of immune response related proteins [130]. Tripartite motif (TRIM) protein family is a large kind of RING-type E3 ligases subfamilies. It participates in regulating numerous cellular activities, especially innate immune responses [131]. As previously revealed, the TRIM family members may not only be a potential viral restriction factors, but also have some anti-viral functions, suggesting their roles in immune response function [132, 133]. TRIM25 targets the N-terminal CARDs of the viral RNA receptor Retinoic-acid-inducible gene-I (RIG-I). RIG-I interacts with MAVS for K63-linked ubiquitination, inducing the activation of type I interferon-mediated host protective innate immunity against viral infection [106, 107]. TRIM25 may also target tumor suppressor P53 for ubiquitination and degradation in response to anti-tumor immunity. Because p53 can promote the expression of interferon-stimulated genes (ISGs) through upregulation of IRF9, a component of the ISG factor 3 (ISGF3) [108, 109]. Besides, TRIM25 is capable of promoting prostate cancer cell proliferation via modulating P53 signals [110]. The RING type E3 ligase RNF2 is a potential interferon-dependent antiviral responses inhibitor. RNF2 inhibits type-I-interferon-mediated antiviral response through directly binding to STAT1, thereby increasing its K33-linked polyubiquitination to separate STAT1/STAT2 from DNA [134, 135]. Immune checkpoints are definitely important for immune system to inhibit abnormal and excessive immune response and avoid self-damage [136]. Unfortunately, tumors can even utilize the immune checkpoints pathway to escape the immune surveillance and anti-tumor immune response, thus to promote tumor growth and progression [137, 138]. Programmed cell death protein 1 (PD-1) and its ligand PD-L1 have been proved to be one of the efficient immune checkpoints targets for treating human cancers. Their aberrant expression may inhibit T cell effector activity and promote tumor immune escape [139]. PD-1 also undergoes multiple post translational modifications. Ubiquitination is essential in maintaining the stability of PD-1 [111]. Furthermore, many E3 ligases have been reported to regulate PD-1 homeostasis. For instance, FBXO38 is the specific E3 ligase of PD-1 that promotes the degradation of PD-1 through K48-linked polyubiquitination in 293T and Jurkat cells. FBXO38 is important for controlling the anti-tumor activity of T cells by regulating the expression of PD-1 [111, 112]. The E3 ligase KLHL22 interacts with PD-1 and promotes the ubiquitination degradation of PD-1 before transportation to the cell surface, thereby enhancing tumor immunity [140]. The RING type E3 ligase Casitas B lymphoma (c-Cbl) regulates the expression of PD-1/PD-L1 in colorectal cancer. C-Cbl targets PD-1 for ubiquitination degradation through the interaction between the C-terminus of c-Cbl and the cytoplasmic tail of PD-1. C-Cbl also inhibits PD-1 by inactivating PI3K/Akt, Jak/Stat, and MAPK-Erk signaling (Table 1) [113].

### Regulation of metabolism

Metabolism is the general term for all chemical changes in organisms that is complex and unified, mainly including glucose metabolism, lipid metabolism, amino acid metabolism [141]. Metabolic changes are one of the important characteristics of tumors. In order to maintain continuous proliferation, tumor cells must adjust their metabolism and nutrient acquisition methods, such as Warburg effect [142, 143]. Moreover, as an important PTM, ubiquitination can also participate in regulating metabolic pathway, indicating that E3 ligases may be involved in regulating tumor metabolism. Peroxisome proliferator-activated receptors (PPARs) are key regulatory factors in response to lipid metabolism, containing PPARα, PPARγ, and PPARβ/δ [144]. PPARγ is an E3 ligase and could degrade nuclear factor κB (NFκB)/p65 via ubiquitination, resulting in the inhibition of NFκB-mediated inflammatory responses and tumor growth [145]. PPARα can be monoubiquitinated by E3 ligase MuRF1 to modulate its localization. PPARα also interacts with E3 ligase MDM2 to regulate the transcriptional activity [146, 147]. Additionally, PPARα can interact with progestin and adipoQ receptor 3 (PAQR3) to promote the ubiquitination mediated degradation of PPARα by the E3 ligase HUWE1. Therefore, such degradation affects the role of PPARα in lipid metabolism [148]. As an important component of the SCF complex, Skp2 triggers the ubiquitination of Akt, which is critical for the regulation of Warburg effect [149]. In addition, Skp2 can also influence cell glucose uptake and glycolysis through Akt ubiquitination [94, 150, 151]. The U-box E3 ligase carboxyl terminus of Hsc70-interacting protein (CHIP) inhibits ovarian cancer progression by suppressing aerobic glycolysis. CHIP targets the tumor glycolysis regulator pyruvate kinase isoenzyme M2 (PKM2) for proteasome degradation [114]. The heterogeneous microenvironments are highly related with solid tumors. Hypoxia is one of the most well studied microenvironments associated with solid tumor development. Hypoxia can also affect the increased chemoradiotherapy resistance and is critical for tumor metabolism [152]. Hypoxia-inducible factor 1 (HIF-1), the key protein in response to hypoxia, is a heterodimeric protein consisting of two proteins — HIF-1α and HIF-1β. HIF-1α is a transcription factor. The nuclear translocation of HIF-1α can promote the transcription of many genes involved in tumor cell glucose metabolism, such as GLUT1, PDK1, and LDHA [153]. The tumor suppressor Von Hippel-Lindau (VHL) is one of the best-known E3 ligases for HIF1α in normoxia. VHL can target the proline hydroxylation modified HIF-1α for ubiquitination and degradation via 26S proteasome. VHL can inhibit the transcriptional function of HIF1α to glucose metabolism associated genes in various cancers especially renal cancer [115–117]. Metabolic pathway consists of various key metabolic enzymes, most of which may also undergo ubiquitination modification. Hexokinase 2 (HK2), the first enzyme in the glycolytic pathway, is highly related to cancer progression. HK2 is prone to be recognized by the autophagy receptor protein SQSTM1/p62 for autophagic degradation after K33-linked polyubiquitination by the E3 ligase TRAF6 in liver cancer [154]. Phosphoinositide-dependent protein kinase 1 (PDK1) is also one of the most important metabolic enzymes, which plays crucial roles in cancer signaling pathways, especially PI3K/Akt and Ras/MAPK pathways [118, 119]. Additionally, the expression or activity of PDK1 can also be aborted by E3 ligases. The E3 ligase RNF126 has been found to target PDK1 for proteasomal degradation to promote cancer cell progression [104]. The small molecule ubiquitin protein UFM1 is reported to increase the degradation of PDK1 via ubiquitination, resulting in the inhibition of PI3K/Akt signaling in gastric cancer development (Table 1) [120].

## Target E3 ligase as a novel therapeutic approach in cancers

E3 ubiquitin ligases can affect most aspects of eukaryotic biological processes by promoting protein ubiquitination and degradation [155, 156]. Both the occurrence and progression of tumors are accompanied by abnormalities in the ubiquitin system [157]. Therefore, the clinical success of proteasome inhibitors makes it possible to target the UPS for developing a series of diagnostic and therapeutic methods against tumors [6]. As the usual proteasome inhibitor, bortezomib or MG132 blocks the degradation of entire proteins, however, drugs targeting a specific E3 ligase may have better selectivity with less toxicity [158].

In order to target specific E3 ligases for cancer drugs development, the RING type MDM2 (murine double minute 2) could be the first choice due to its overexpression in various human cancers [159, 160]. MDM2 is a direct downstream target of the genome guardian protein p53, which regulates the function and expression of many important genes related with cell cycle arrest, DNA repair, and apoptosis [161]. MDM2 tightly interacts with p53 for its ubiquitination and proteasomal degradation [162]. Due to both of its oncogenic potential and negative regulation of p53, MDM2 is thought to be a striking and meaningful drug target for cancer therapy. Therefore, many small molecules that inhibit MDM2 have been designed [163]. By using high-throughput screening, the potential small molecules Nutlins, a family of cis-imidazoline analogues, have been identified and tested in clinical trials. These are the first small molecule inhibitors designed to bind to MDM2, thereby disrupting its interaction with p53 [164, 165]. There are also many other small molecules identified, which break the interaction of MDM2 and p53. For example, MI-219 can target MDM2 to disrupt its interaction with p53, promoting cancer cell cycle arrest and selective apoptosis [166]. Compared to MI-219, another promising small molecule RITA (reactivation of p53 and induction of tumor cell apoptosis) can prevent the MDM2-p53 interaction by binding p53 instead of MDM2, suggesting that it might block many other possible interactions of p53. Thus, RITA might affect the ubiquitination of p53 and promote the activation of p53 function in tumors [167, 168]. RG7388 (idasanutlin), a second-generation MDM2 inhibitor, was designed to reduce the potency and toxicity profile of earlier small molecules nutlins, which demonstrated a dose-dependent p53 stabilization, apoptosis, and cell cycle arrest during trials [169, 170].

The largest E3 ligases family SCF (Skp1–cullin–F-box proteins) mediates more than 20% ubiquitinated proteins for 26S proteasome degradation, Thus, the F-box protein Skp2, a member of the SCF family, can be another important drug target [171]. The E3 ligase Skp2 has been reported to overexpress in many human cancers and can regulate tumorigenesis, further supporting that Skp2 is a possible target for tumor drugs development [172]. Skp2 inhibitors can be designed from many aspects, such as reducing the expression of receptor part (Skp2) or blocking its interactions with Skp1 (adaptor bridge) or even the target substrates [173]. The E3 ligase SCF-Skp2 conjugated with SKP1 and its accessory protein Cks1 to promote cancer cell proliferation mainly through ubiquitination and degradation of the cyclin-dependent kinase (CDK) inhibitor p27 [174, 175]. Skp2 Inhibitor C1 (SKPin C1) can suppress the Skp2-mediated p27 degradation by disrupting p27 binding through key compound-receptor contacts [176]. Besides targeting p27, Skp2 can also block p53-mediated apoptosis by competing with p53 for the binding of p300, a transcriptional coactivator. Thus, Skp2 inhibits p300-mediated p53 acetylation [177, 178]. In addition, Skp2 is an important negative regulator of p53 that is overexpressed in many aggressive cancers. Hence, inhibition of the Skp2/p300 protein–protein interaction (PPI) for re-activating p53 may be an attractive target for cancer treatment [173, 178]. The Skp2 inhibitor M1 disrupts the p300-binding site of Skp2, thereby releasing p300 to bind p53 for acetylation and increasing p53-mediated apoptosis [179]. The Skp2 inhibitor SMIP004 is able to increase the curative effect of tumor radiotherapy. SMIP004 can even decrease the protein stability of PCAN, which is the target of Skp2, and inhibit breast cancer cell proliferation (Table 2) [86].

**Table 2:** Description of the classifications, Pros and Cons between PROTACs and small molecule inhibitors (SMIs)

Types	Classifications		Pros	Cons	Refs
	1.proteasome inhibitors: bortezomib, MG132		1. Generally, cell and tissue permeable and high oral bioavailability 2. In some situations, more tolerable	1.More dosing to reach the therapeutic concentration causing more toxicities 2.Incapable of targeting undruggable and mutated proteins	[158]
**SIMs**	2.E3 ligases inhibitors: a. MDM2 targeting drugs: Nutlins, MI-219, RITA, RG7388 b. SKP2 targeting drugs: SKPin C1, Skp2 inhibitor M1, SMIP004				[164–170] [86, 176, 179]
	**E3 ligands**	**POI ligands**	1.More potent and longer lasting effect with lower concentration and lower toxicities 2.Capable of undruggable and mutated proteins 3.Overcome resistance to SMIs 4.Possible of tumor selectivity	1.Less cell and tissue permeable and more challenges for oral administration due to its high molecule weight 2.Undesirable toxicities 3.Complete degradation of specific proteins and degradation of undesirable proteins	
	MDM2-based	AR, BRD4			[180, 181]
**PROTACs**	IAP-based	CRABP-1/2, ERα			[168, 169]
	CRBN-based	BETs, BRD4			[171, 172]
	VHL-based	HIF1α, AR, ERRα, BCL-XL, BRD4			[175, 182]

**Fig. 5:**
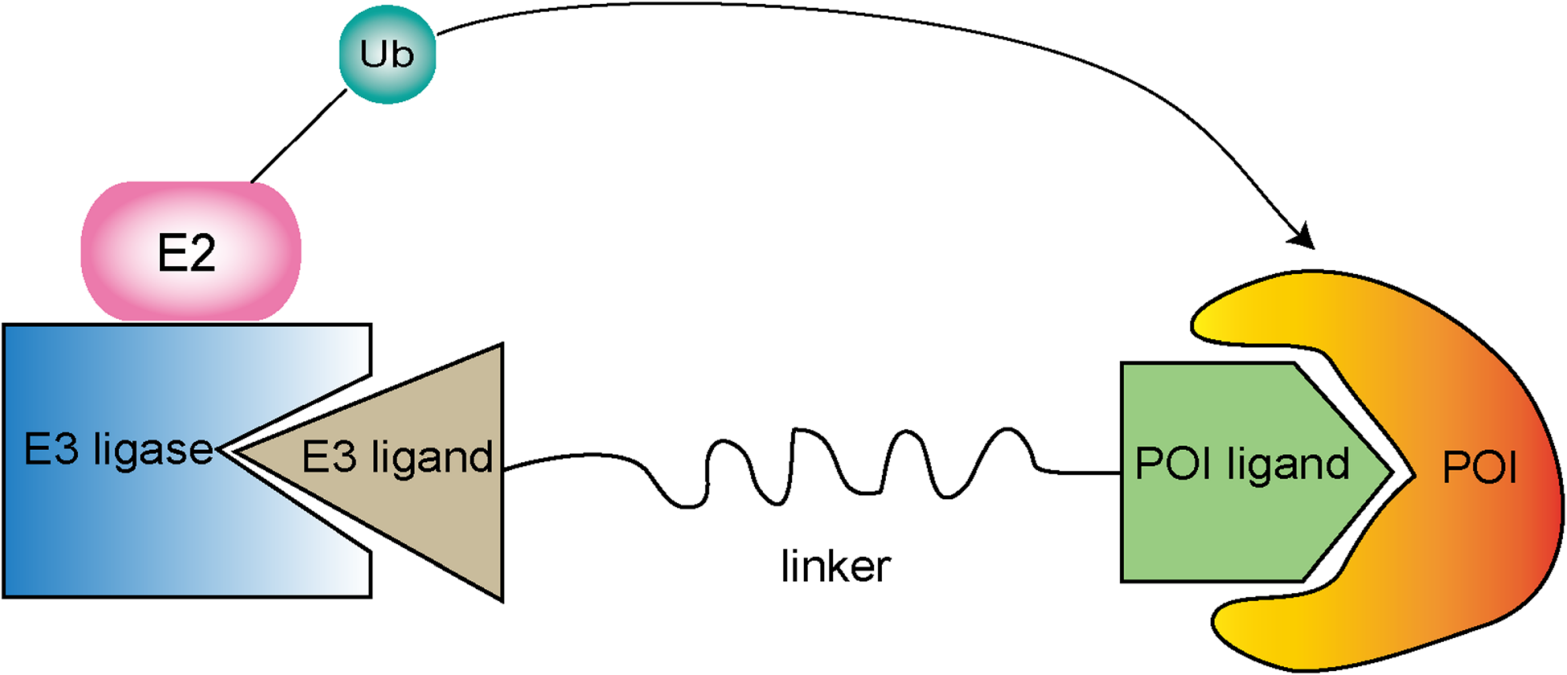
Schematic diagram of PROTACs. A Graphical representation of the components and process of PROTACs. The PROTACs consist of three important parts, including a ligand binding to the POI, a covalently linked ligand of an E3 ubiquitin ligase and a linker to link these two ligands

Importantly, it is noteworthy that the emerging technology proteolysis-targeting chimeras (PROTACs) have potential advantages compared with traditional small molecule inhibitors (SMIs). PROTACs can bind to the target protein and recruit E3 ubiquitin ligase, so that the target is labeled with ubiquitin and further degraded by the 26S proteasome [182]. PROTACs are bifunctional molecules composed of two distinct ligands. One is designed for binding the protein of interest (POI), and the other is covalently linked and for the specific E3 ligase binding (Fig.5) [183]. It has been nearly 20 years since the concept of PROTAC proposed by the Crews and his colleagues in 2001. The first PROTAC was designed to recruit the SCF E3 ligase β-TRCP to target the POI methionine aminopeptidase-2 (MetAp-2) for degradation [184]. So far, there are more than 50 proteins targeted by PROTACs, some of which have been proved to be clinical drug targets for cancer therapy [183]. Importantly, four kinds of E3 ligases are commonly chosen for PROTACs, including MDM2, inhibitor of apoptosis proteins (IAPs), cereblon (CRBN) and VHL [185]. The first all-small molecule PROTAC utilizes nutlin to recruit the E3 ligase MDM2 to degrade androgen receptor (AR) in prostate cancer cells [180]. In 2019, a new MDM2-based PROCTAC A1874 is designed to target bromodomain-containing protein (BRD4) for degradation and stabilize the tumor suppressor P53 to inhibit cancer progression simultaneously [181]. In 2010, Y. Hashimoto and his groups successfully linked methyl bestatin (MeBS) with different length all-trans retinoic acid recruiting E3 ligase IAPs to degrade retinoic acid-binding proteins (CRABP-1/2) [186]. The IAP-based PROTAC named Specific and Non-genetic IAP-dependent Protein Eraser (SNIPER) is designed to target various proteins, especially estrogen receptor alpha (ERα), for degradation in breast cancer [187]. Additionally, the E3 ligase CRBN is the target of many drugs, such as thalidomide and its analogs. Therefore, many PROTACs based on CRBN against multiple targets have been designed [188]. The first CRBN-based PROTAC mainly targets BET proteins in acute myeloid leukemia (AML) [189]. Another CRBN-based against BET PROTAC named ARV-825 utilizes the link between OTX015 and E3 ligase CRBN to promote BRD4 degradation, eventually inhibiting the cancer cell progression [190]. Strikingly, there are also many other inhibitors based on PROTACs technology, such as ARV-471 and ARV-110, which are both under phase I clinical trials. ARV-471 is ERα-targeted PROTAC against breast cancer progression [191]. ARV-110 is AR-targeted PROTAC against prostate cancer [192]. The E3 ligase VHL is one of the most popular targets for PROTACs. As described previously, VHL E3 ligases complex is essential for regulating the expression of HIF1α that contributes to various cancers development. So far, many kinds of inhibitors, especially PROTACs targeting VHL for HIF1α degradation, have been designed [193]. The VHL-based PROTAC named ARD-266 can also target AR for degradation in prostate cancer [194]. As reported, VHL-based PROTAC is able to regulate cellular energy homeostasis by degrading estrogen-related receptor alpha (ERRα), which is extremely important during mitochondrial biogenesis [195]. The inhibitor DT2216 developed by PROTAC technology is discovered to show enhanced anti-tumor potency by targeting E3 ligase VHL to promote BCL-XL degradation [196]. MZ1 is an efficient compound using PEG as a linker to tether pan-BET selective bromodomain inhibitor JQ1 to VH032, a potent and specific VHL ligand. Thus, MZ1 can target BRD4 for degradation in cervical cancer (Table 2) [197, 198].

Above all, E3 ligases are important and efficient target for cancer therapy. Till now, each kind of drugs based on E3 ligases has both benefits and drawbacks. The PROTACs technology shows high selectivity, specificity and potential to target undruggable proteins.

## Conclusions and perspectives

From this review, we have briefly illustrated the principles of ubiquitin system and the important role of E3 ligases in cancer progression. The E3 ubiquitin ligases can modulate various biological development processes, including cell proliferation, apoptosis, DNA damage repair, immunity, and metabolism. It’s worth noting that E3 ligases can function either as tumor promoters or suppressors. And unlike many other elements in the UPS (e.g. E1, E2), E3 ligases are not only large in quantity but also directly target specific substrates for degradation. Thus, E3 ligases can be a promising and effective target for cancer therapy. It is a great expectation that targeting specific E3 ligases would induce apoptosis or sensitize cancer cells to apoptosis induced by conventional anti-cancer therapies. In contrast with targeting a specific E3 for inhibition, the promising technology PROTACs is more potent in many cases. Although, many small molecule inhibitors and PROTACs have been tested in cellular experiments or even clinical trials, both of these two approaches have their own disadvantages. Therefore, it is still a long way to put in use for a novel class of anticancer drugs, as well as discover and develop more efficient E3 ligases targeted inhibitors.

## Data Availability

Not applicable.

## Abbreviations

USP: Ubiquitin proteasome system
PTM: Posttranslational modification
HECT: Homologous to the E6AP carboxyl terminus
RING: Really interesting new gene
CRLs: Cullin RING ligases
APC/C: Anaphase-promoting complex/cyclosome
RBR: RING-IBR-RING
LUBAC: Linear ubiquitin chain assembly complex
DSB: DNA double-strand break
NHEJ: Non-homologous end joining
HR: Homologous recombination
RIG-I: Retinoic-acid-inducible gene-I
ISGs: Interferon-stimulated genes
PD-1: Programmed cell death protein 1
c-Cbl: Casitas B lymphoma
PPAR: Peroxisome proliferator-activated receptors
CHIP: Carboxyl terminus of hsc70-interacting protein
PKM2: Pyruvate kinase isoenzyme M2
HIF-1: Hypoxia-inducible factor 1
VHL: Von hippel-lindau
HK2: Hexokinase 2
PDK1: Phosphoinositide-dependent protein kinase 1
SCF: Skp1–cullin–F-box proteins
SMIs: Small molecule inhibitors
PPI: Protein–protein interaction
PROTACs: Proteolysis-targeting chimeras
POI: Protein of interest
MDM2: Mouse double minute 2 homolog
RITA: Reactivation of p53 and induction of tumor cell apoptosis
IAPs: Inhibitor of apoptosis proteins
CRBN: Cereblon
BRD4: Bromodomain-containing protein
BET: Bromodomain and extra-terminal
ERRα: Estrogen-related receptor alpha
SNIPER: Specific and non-genetic IAP-dependent protein eraser
